# Neuroimmune Pathophysiology of Long COVID

**DOI:** 10.1111/pcn.13855

**Published:** 2025-06-19

**Authors:** Janna K. Moen, Christopher A. Baker, Akiko Iwasaki

**Affiliations:** 1 Howard Hughes Medical Institute, Chevy Chase, MD; 2 Department of Immunobiology, Yale University School of Medicine, New Haven, CT; 3 Center for Infection and Immunity, Yale University School of Medicine, New Haven, CT

## Abstract

Although COVID-19 was originally considered a respiratory illness, it is now well established that SARS-CoV-2 infection can have far-reaching impacts on the nervous system. Neurological symptoms such as chemosensory dysfunction are frequently observed during acute infection and approximately 10% of COVID-19 cases will go on to develop new or persistent long-term symptoms, a condition known in the literature as post-acute symptoms of COVID-19 (PASC) or by the patient-coined term Long COVID. Common neurological symptoms in Long COVID include new onset cognitive difficulties, dysautonomia, fatigue, and peripheral neuropathy. The emergence of Long COVID has prompted renewed interest in the study of post-acute infection syndromes (PAIS), particularly in the area of neuroimmune interactions. In this Review we provide a comprehensive overview of the current body of literature on neurological manifestations of SARS-CoV-2 infection and Long COVID, with an emphasis on neuroimmune mechanisms drawn largely from autopsy studies and animal models. A more complete understanding of neuroimmune crosstalk in Long COVID will not only guide the development of therapies for this highly disabling condition but will also contribute to our general understanding of neuroimmune interactions in health and disease.

## Introduction to Long COVID

SARS-CoV-2 infects human hosts via the viral entry receptor angiotensin-converting enzyme 2 (ACE2) expressed in epithelial cells throughout the body. Infection by SARS-CoV-2 results in heterogeneous outcomes in human hosts, from asymptomatic, mild, moderate, severe to lethal COVID-19. Despite reductions in the frequency of severe COVID-19, a substantial percentage of patients develop new or persistent chronic symptoms weeks or even months after their infection. The term “Long COVID” was originally coined by patients to describe the chronic illness state that followed an acute COVID-19 infection and encompasses many different presentations and phenotypes, with over 200 symptoms now associated with the condition.^1–4^ The impact of Long COVID also varies considerably, ranging from mild impairment to severe disability. The prevalence of Long COVID following SARS-CoV-2 infection is reported to be around 10% internationally,^5–7^ although the highly heterogeneous symptoms and lack of consistent case definitions complicate frequency estimates. The term can be inclusive of various phenotypes including individuals with post-intensive care syndrome, those with a prolonged recovery from SARS-CoV-2, patients with new symptoms and conditions that emerge following an asymptomatic or mild infection, and patients whose underlying health conditions were exacerbated by infection. Long COVID can impact patients of any demographic, including children and young adults.

While any SARS-CoV-2 infection can precipitate Long COVID, the epidemiology of the condition is impacted by several features of the acute infection. Patients with severe COVID-19 have a high incidence of lingering symptoms,^8,9^ likely as a direct consequence of the high levels of inflammation and cytokine storm present in severe disease. Both vaccination and previous infections reduce the incidence of severe COVID-19, but the continued evolution of novel SARS-CoV-2 variants alongside its ability to evade host immunity means that patients are still vulnerable to breakthrough infections or reinfection events. As a result, the majority of patients presenting with Long COVID experienced a mild or moderate SARS-CoV-2 infection.^10^ Furthermore, repeated infections may increase the risk of long-term sequelae^11^ and self-reported Long COVID symptoms,^12,13^ although some studies have found decreased risk of new-onset Long COVID following reinfection.^14,15^ Viral strain may also impact the course of both acute infection and Long COVID as accumulated mutations impact how the SARS-CoV-2 virus interacts with host tissues. Strain differences in neurotropism and virulence have been reported in rodent models^16–18^ and in human stem cell-derived sensory neurons.^19^ As different viral strains evolved alongside the development of novel therapeutics and vaccine strategies to target SARS-CoV-2, strain differences in Long COVID can be challenging to study at the epidemiological level. One meta-analysis from 2022 reported that the frequency of specific symptoms differed by variant, although each variant examined (wild-type, Alpha, Delta, and Omicron) was capable of inducing persistent symptoms.^20^ Similarly, an Italian observational multicenter study reported no significant differences in the prevalence of Long COVID symptoms throughout the different pandemic waves.^21^

The highly varied disease profile makes Long COVID a particularly challenging entity to both study and treat in the clinic. Several risk factors have been identified which include female sex and pre-existing comorbidities;^8,9,22^ additionally, several studies have identified genetic polymorphisms associated with Long COVID.^23–26^ A substantial number of patients with Long COVID present with an illness resembling myalgic encephalomyelitis/chronic fatigue syndrome (ME/CFS),^27,28^ a neuroimmune disorder of unknown pathophysiology often precipitated by an infection event and characterized by a severe worsening of symptoms following minimal physical or mental exertion. ME/CFS is highly disabling with no effective treatments and a poor prognosis, as less than 10% of patients report recovery after several years of illness.^29^ Several studies have demonstrated overlapping symptomology and biological perturbations in Long COVID-ME/CFS cases and non-COVID ME/CFS,^30^ suggesting the two conditions may share some underlying mechanisms.

In 2024, the National Academies of Sciences, Engineering, and Medicine (NASEM) published a consensus definition of Long COVID developed with patient, stakeholder, and research expert participation. They define Long COVID as “an infection-associated chronic condition that occurs after SARS-CoV-2 infection and is present for at least 3 months as a continuous, relapsing and remitting, or progressive disease state that affects one or more organ systems.”^31^ This definition is intentionally inclusive of various symptom presentations and emphasizes that Long COVID is a complex disorder that cannot be easily diagnosed through laboratory testing. Without definitive biomarkers, it can be difficult to determine whether a patient’s symptoms are attributed to Long COVID. Clinicians are encouraged to refer to the NASEM report for detailed discussion of the recommended diagnostic process for Long COVID.^32^

Considering the high frequency of Long COVID, continued community SARS-CoV-2 transmission alongside waning immunity and high rates of reinfection means that Long COVID represents a serious and growing public health crisis. Neurological manifestations are frequently reported in both acute COVID-19 and Long COVID, bringing renewed attention to the study of neuroimmune interactions in infectious diseases. In this Review, we provide a narrative overview of the nervous system in COVID-19 and how the immune system contributes to the pathophysiology of neurological Long COVID. Proposed mechanisms and systemic neuroimmune dysfunction are covered, followed by a detailed discussion of potential mechanisms of immune-mediated pathologies within specific divisions of the nervous system.

## Proposed mechanisms of Neuro-Long COVID

While the neurological impacts of COVID-19 have been well described, the mechanisms driving these disease processes are still unclear. At least four mechanisms have been proposed, each of which may be present to various degrees across different patient subsets:

### Neuroinvasion.

Although SARS-CoV-2 was originally considered to be a respiratory virus, it appears capable of infecting various cell types and organs, including the nervous system. Infection of the nervous system has been demonstrated in early infection in autopsy studies,^33–36^ cultured brain cells and organoids,^35,37–40^ and animal infection models.^16–18,35,41–43^ However, other studies have failed to observe any evidence of direct viral infection in brain tissue from patients who died of COVID-19,^44–46^ brain tissue biopsies from surgical patients with perioperative mild to moderate COVID-19,^47^ or cerebrospinal fluid from acute and Long COVID patients.^48^ These contradictory findings may be attributable to the technical challenge of detecting viral infection in human post-mortem tissues, which often involve long or variable tissue processing intervals. The prevalence and extent of direct neuroinvasion remains unclear and is likely influenced by multiple factors, including infection severity and viral strain.

SARS-CoV-2 appears capable of infecting various cell types in the nervous system including neurons, astrocytes, and epithelial cells. ACE2 and other coronavirus coreceptors can be observed in the brain but the precise localization of ACE2 within the central nervous system remains controversial. Neurons derived from human pluripotent stem cells can express ACE2 and facilitate infection *in vitro*,^39,49^ although most studies using human or rodent brain tissue find that ACE2 protein is either undetectable in neurons^50,51^ or is localized to discrete nuclei.^38,39,52,53^ *In vitro*, however, SARS-CoV-2 can directly infect neurons and disrupt synaptic transmission, for example by promoting cellular fusion or cellular senescence.^54–56^ The extent to which this occurs in human patients is unknown, although direct infection of the olfactory bulb and some cortical areas has been reported in non-human primates.^43,57^ Alternatively, human and animal studies have reported more robust ACE2 expression in astrocytes, pericytes, and endothelial cells of the choroid plexus and blood-brain barrier.^39,50–52,58^ As SARS-CoV-2 does not appear to be capable of transsynaptic infection within neural circuits,^59^ the virus may preferentially target epithelial cells in the nasal mucosa,^34,59^ choroid plexus,^58,60^ or nervus terminalis neurons in the olfactory epithelium^61,62^ to propagate within the nervous system. Furthermore, infection of olfactory neurons in the absence of ACE2 expression might be explained by the formation of tunneling nanotubes between infected epithelial cells and neurons.^63^ Determining the extent of direct neural infection is complicated by strain-specific effects widely reported in animal studies.^16–18,41^

### Persistent inflammation and tissue damage.

COVID-19 is a highly inflammatory disease, and a wealth of literature has shown that peripheral immune responses may be sufficient to drive neuroinflammation and dysfunction even in the absence of direct brain infection or injury. Widespread dysfunction in circulating immune cells has been repeatedly demonstrated in both acute COVID-19 and Long COVID.^64^ Patients who died from severe COVID exhibit brain-wide CD8+ T-cell infiltration,^65^ and Long COVID patients show elevated plasma levels of IL-1β, IL-6, and TNFα 8–10 months post-infection.^66^ One report found substantial perturbations in T cell subsets of Long COVID patients, including increased frequencies of migratory CD4+ cells and exhausted SARS-CoV-2-specific CD8+ cells,^67^ suggesting a sustained adaptive immune response that may contribute to immune dysregulation and promote inflammation in impacted tissues. Similarly, a recent study from the same group used a specialized PET tracer to detect activated T lymphocytes and reported increased T cell activation in multiple tissues of post-COVID patients, including the brainstem and spinal cord.^68^ These data highlight ongoing T cell infiltration of the central nervous system in post-COVID participants, which could reflect a combination of immune dysregulation and blood-brain barrier disruption. Additionally, a prospective cohort study examining CSF and serum from infected individuals found unique antibody isotype profiles in the CSF of those who developed long-term neurological symptoms.^69^ Long COVID has also been linked to genetic polymorphisms in genes of the immune system,^23–26^ further supporting a role of immune dysregulation in disease pathogenesis. Collectively, persistent inflammation can damage neural tissues leading to neurological symptoms.

### Viral RNA and antigen persistence.

Reservoirs of persistent viral RNA or antigen could contribute to chronic inflammation and tissue dysfunction after infection.^70^ Autopsy studies have detected persistent SARS-CoV-2 RNA in multiple organs, including the brain, at least 230 days following infection,^36^ and non-human primates infected with SARS-CoV-2 show persistent viral mRNA in pyriform cortex and amygdala at 28 days post-infection.^71^ Persistent viral RNA is expected to stimulate innate immune responses via induction of inflammatory cytokines and type I interferons, leading to activation and recruitment of T and B cell-mediated adaptive immunity. In the event that viral RNA persists within the nervous system, the resulting chronic inflammatory state can drive neuron damage and dysfunction. Even in the absence of direct viral infection and replication, circulating fragments of the SARS-CoV-2 virus can have profound impacts on the nervous system.^72^ Persistent SARS-CoV-2 proteins, particularly the S1 spike protein, are frequently reported in the blood of Long COVID patients (and often observed in a smaller percentage of convalescent controls).^73–76^ SARS-CoV-2 infection has been established to disrupt the blood-brain barrier in both acute infection and Long COVID,^72^ potentially allowing circulating viral antigens access to the central nervous system. As direct delivery of SARS-CoV-2 proteins to the brain is sufficient to induce neuroinflammation and neuronal dysfunction in rodent models,^77–82^ persistent viral proteins in the blood may be able to drive neuropathology independently. Persistent spike protein was also recently demonstrated in the skull and meninges of COVID-19 patients long after viral clearance, a result that was replicated in mice injected with spike protein.^83^

### Autoimmunity.

COVID-19 infection can induce autoimmunity in adult patients^18,84,85^ and in the emergent Multisystem Inflammatory Syndrome in Children (MIS-C) that is precipitated by COVID-19.^86^ Smaller studies have demonstrated higher prevalence of adrenergic and muscarinic receptor autoantibodies measured by ELISA^87^; another report found anti-neuronal antibodies in the CSF of 52% of neurological Long COVID patients, albeit without a control comparison group^88^. These findings are in line with retrospective cohort studies and meta-analyses that report increased incidence and risk of autoimmune disorders in COVID-19 survivors.^89,90^ Additionally, two recent preprints demonstrate that IgG immunoglobulins isolated from Long COVID patients can induce symptoms in mice by targeting neuronal tissues,^91,92^ supporting a role of autoimmunity in the pathogenesis of Long COVID. There is also speculation that viral surface glycoproteins in SARS-CoV-2 infection could initiate the development of autoantibodies against the structurally similar myelin-associated glycoprotein.^93^ Autoreactive antibodies and T cells targeting neural tissues can damage or alter target cell function, leading to neurological dysfunction and neurodegeneration.

## Systemic neuroimmune dysfunction in Long COVID

### Neuroinflammation

**Fig. 1 –:**
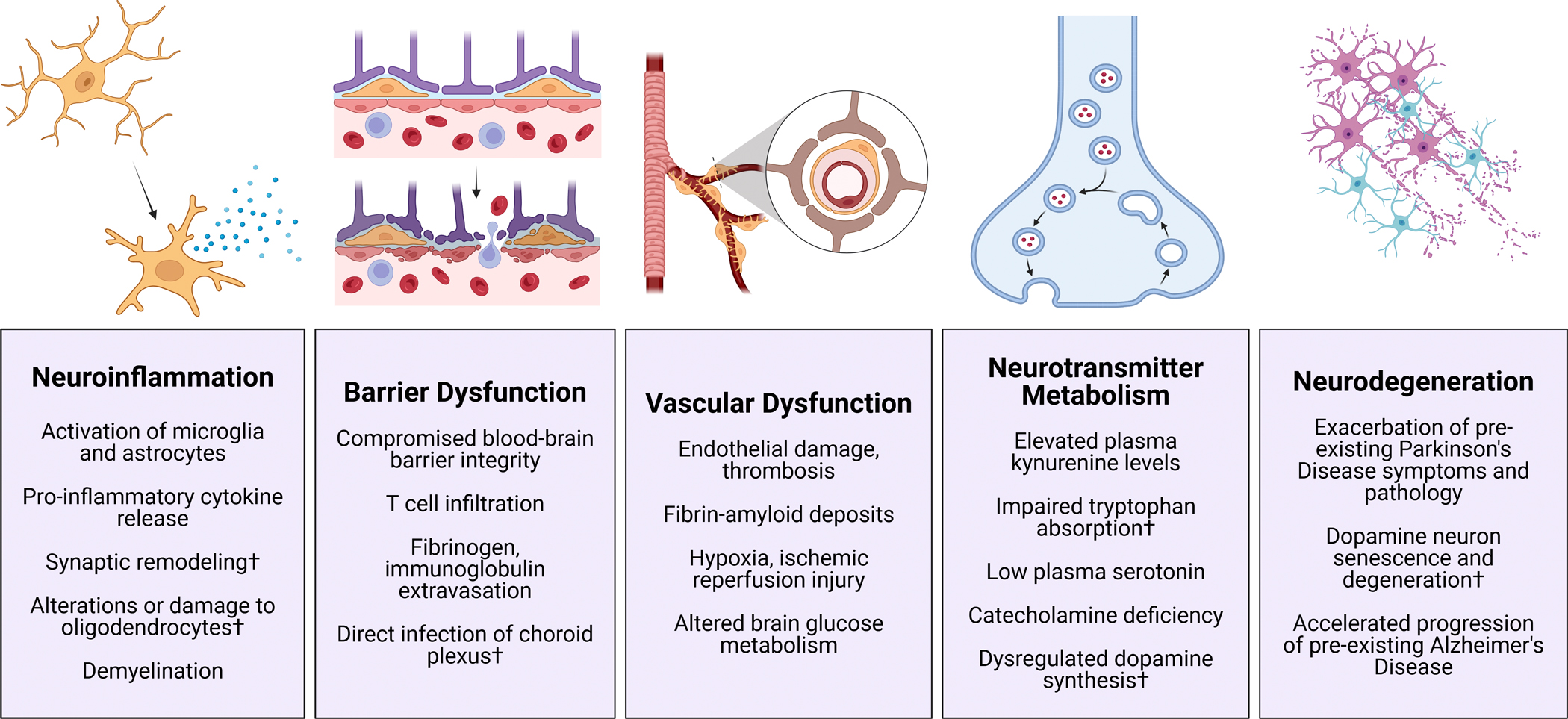
Systemic neuroimmune manifestations of Long COVID demonstrated in the reviewed studies. † denotes results from autopsy studies, animal models, or *in vitro* experiments that have not been directly measured in Long COVID. Figures generated using Biorender.

The central nervous system (CNS) was traditionally considered to be “immune privileged” due to the presence of the blood-brain barrier, which limits recruitment of T-cell-mediated adaptive immunity.^94^ Instead, resident microglia and macrophages make up the immune network in the CNS parenchyma. Upon immune stimulation, these cells take on a pro-inflammatory phenotype that includes the production and release of inflammatory cytokines as well as synaptic remodeling via phagocytosis.^95^ This neuroinflammation appears to play a prominent role in the pathogenesis of both acute and Long COVID (fig. 1). Activated microglia accumulate throughout diverse areas of the CNS in COVID-19 postmortem studies^96–101^ and animal models of SARS-CoV-2 infection.^57,102–106^ Intra-cisterna magna (ICM) infusion of spike protein^80,81^ or envelope (E) protein^77^ are sufficient to induce neuroinflammation and behavioral deficits in rodent models, suggesting that SARS-CoV-2 proteins may drive specific pathology in the nervous system without requiring direct viral invasion or replication. Moreover, ICM infusion of the S1 spike domain potentiated neuroinflammatory responses to LPS in mice, suggesting S1 could “prime” the neuroimmune network response to subsequent inflammatory stimuli.^82^ These observations led to speculation that COVID-19 infection may trigger a state of chronic neuroinflammation in Long COVID patients, who often report flu-like symptoms that resemble prolonged sickness behaviors. Positron emission tomography (PET) studies demonstrate higher binding levels of a radioligand targeted against the TranSlocator PrOtein (TSPO), a molecule that is upregulated in pro-inflammatory microglia and macrophages, in the brains of Long COVID patients.^107,108^ Consistently, SARS-CoV-2 infection in a brain organoid model induced synapse loss by increasing microglial engulfment of postsynaptic terminals.^109^

In addition to microglia, SARS-CoV-2 infection impacts other glial cells of the nervous system. Astrocytes play a crucial role in maintaining homeostasis in the CNS environment and can also enter a heightened “active” state in response to inflammatory stimuli, which can be observed in acute COVID-19 and Long COVID.^46,96,99,100,110^ These clinical data align with *in vitro* work demonstrating that direct exposure of brain organoids to SARS-CoV-2 predominantly infects astrocytes, possibly through less traditional coronavirus coreceptors (i.e. CD147, DPP4).^37^ Oligodendrocytes of the CNS and Schwann cells in the periphery are glial cells responsible for generating and maintaining the conductive myelin sheathes that protect axons and facilitate fast signal propagation. In a mouse model of mild respiratory SARS-CoV-2 infection, mature oligodendrocytes were depleted in the corpus callosum 7 days post-infection which persisted for at least 7 weeks, accompanied by a reduction in the number of oligodendrocyte precursor cells and reduced myelination of axons.^111^ CNS demyelination measured by electron microscopy showed that the pattern was diffuse and not observed in focal lesions, consistent with altered transcriptional states of oligodendrocytes in the brainstem during acute COVID-19^100^ without evidence of demyelinating lesions using MRI.^112^ However, macromolecular proton fraction mapping demonstrated demyelination throughout the brain in Long COVID, particularly in patients with post-COVID depression compared with uninfected and convalescent controls.^113^ This demyelination was not restricted to white matter tracts but was also observed in grey matter structures like the hippocampus, putamen, and globus pallidus. The mechanism and prevalence behind post-COVID oligodendrocyte dysfunction is still unknown but likely involves multiple factors, including actions by activated microglia and astrocytes.^114^

### Neurotransmitter dysfunction

Neuroinflammation can also trigger long-term disruptions in neuronal signaling and neurotransmitter metabolism (fig. 1). SARS-CoV-2 infection has been shown to induce robust expression of indoleamine 2,3-dioxygenase 2 (IDO2),^115–117^ an enzyme that catalyzes the conversion of tryptophan to kynurenine. High levels of kynurenine have been demonstrated in the blood^118–121^ and urine^122^ of patients hospitalized with COVID-19 and correlate with disease severity, while other reports have found elevated kynurenine metabolite levels in Long COVID patients^116,120,121,123,124^ with some notable exceptions.^125^ Circulating kynurenine readily crosses the blood-brain barrier, and its metabolites include both neurotoxic (quinolinic acid) and neuroprotective (kynurenic acid) molecules.^126^ Quinolinic acid acts as an agonist at synaptic glutamatergic NMDA receptors to potentially drive excitotoxicity.^127^ Dysregulation of this pathway also impacts production of cellular energy in the form of NAD+, which can further exacerbate the oxidative stress that accompanies excitotoxicity.^126^

In further support of dysregulated tryptophan metabolism in COVID-19, a translational study by Wong et al^125^ reported circulating serotonin deficiency in both acute and Long COVID patients. Mouse models showed this effect was not specific to SARS-CoV-2 but was driven by the induction of type I interferons following exposure to viral RNA, which suppressed the absorption of dietary tryptophan in the gut. Low serum serotonin levels have been reported in other cohorts of patients with severe COVID-19 that can persist for at least 9 months post-infection,^128,129^ although low circulating serotonin levels may not generalize to every presentation of Long COVID.^130^ These data have renewed interest in using selective serotonin reuptake inhibitors (SSRIs) to treat Long COVID, although it should be noted that SSRIs work by increasing levels of serotonin within the neuronal synapse; circulating serotonin is stored in platelets, and SSRI medications deplete platelet serotonin levels by preventing uptake through the serotonin transporter, which effectively reduces platelet aggregation.^131–134^ As platelet hypercoagulability is observed in both acute COVID-19 and Long COVID,^106,135–139^ this action by SSRIs may contribute to their therapeutic effect and protect against cardiovascular sequelae,^140–147^ and could explain why SSRI therapy appears to reduce the risk of mortality and post-acute sequelae from COVID-19.^148–153^ Moreover, fluoxetine directly inhibits SARS-CoV-2 replication and infectivity in human lung slices without impacting other respiratory viruses, pointing toward a specific antiviral interaction with SARS-CoV-2.^154^ However, SSRIs have also been shown to increase extracellular histamine levels in brain slice preparations by preventing reuptake through monoamine transporters,^155^ suggesting that these medications may exacerbate mast cell dysfunction and localized inflammation.

Catecholamine neurotransmitters such as dopamine (DA) regulate many processes that are implicated in Long COVID, including motor control, wakefulness, motivation, and the autonomic nervous system. Multiple studies report DA neuron dysfunction and degeneration following SARS-CoV-2 infection,^55,156,157^ and patients with neurological Long COVID exhibit signs of catecholamine deficiency in the cerebrospinal fluid (CSF) at one year post-infection.^158^ In animal models, intranasal infection with mouse-adapted SARS-CoV-2 induced loss of DA neurons in the substantia nigra at 120 days post-infection,^156^ and intranasal infection of transgenic mice with ubiquitous human ACE2 expression (AC70-hACE2) reduced expression levels of the D2 dopamine receptor and tyrosine hydroxylase (TH), the rate-limiting enzyme involved in DA synthesis.^159^ Similarly, human stem cell-derived DA neurons infected with B.1 and Delta variants showed reduced intracellular content and extracellular release of DA alongside reduced expression of TH, dopamine decarboxylase, and the dopamine transporter.^160^ Moreover, DA synthesis could be compromised by oxidative stress due to lowered bioavailability of tetrahydrobiopterin (BH4), a necessary cofactor for catecholamine synthesis.^161^

### Barrier dysfunction

SARS-CoV-2 infection appears to impact physiological barriers in the body, including the nervous system (fig. 1). Early studies using microfluidic models reported that S1 protein itself can disrupt the integrity of the blood-brain barrier.^162–164^ These *in vitro* data have been replicated in animal models of acute infection^105,165–168^ and autopsy studies of COVID-19 decedents,^96,169,170^ which report fibrinogen extravasation and T cell infiltration typical of compromised barrier integrity. More recently, a longitudinal MRI study using dynamic contrast-enhanced perfusion reported changes in blood-brain barrier permeability in the frontal white matter and brainstem of Long COVID patients, which was present at 3 months post-infection and remained at 12 month follow-up.^171^ Interestingly, in this study neurocognitive scores using the Cogsafe Brief Battery did not correlate with the degree of permeability, highlighting the discrepancy between standardized cognitive exams and objective nervous system dysfunction. Significant blood-brain barrier disruption has also been reported in rodent models.^105,167,168^ Accumulating evidence also suggests that SARS-CoV-2 infection impacts the choroid plexus and the blood-CSF interface, and the virus readily infects choroid plexus epithelial cells *in vitro*.^58,60,172^ One study reported that approximately half of COVID-19 patients with neurological symptoms exhibited elevated CSF albumin or total protein for at least 30 days alongside elevated inflammatory cytokines, indicative of prolonged blood-CSF barrier dysfunction.^173^ This was further demonstrated by single-nucleus transcriptomics from frontal cortex and choroid plexus in COVID-infected and control individuals, which reported broad cellular perturbations in barrier cells of the choroid plexus alongside peripheral T cell infiltration of the parenchyma.^46^ More recently, an MRI study found increased choroid plexus volume in subacute COVID-19 patients compared with asymptomatic COVID-19, healthy controls, and non-COVID encephalopathy, suggesting a SARS-CoV-2-specific mechanism.^174^ The involvement of the choroid plexus and blood-CSF barrier in Long COVID is less clear, although a recent retrospective study of 84 CSF samples from Long COVID patients reported few major pathologies but found elevated total protein and albumin levels in 24% and 13% of samples, respectively.^175^

### Vasculature and neurovascular coupling

SARS-CoV-2 infection exerts significant effects throughout the body’s vascular system (fig. 1).^138,176^ The prevalence of cardiovascular and cerebrovascular complications is increased following infection,^138,177^ with thrombosis and endotheliitis as well-established consequences of severe COVID-19.^176,178,179^ A 2021 autopsy study of infected patients reported a high prevalence of cerebral hemorrhage and diffuse intravascular thrombosis,^178^ while a translational study comparing autopsy tissue with animal models reported that the main protease of SARS-CoV-2 can induce the death of human brain endothelial cells and cause collapsed capillaries in mice.^180^ Similar findings were reported in a postmortem autopsy study of patients who died during the first wave in 2020: all patients had multifocal vascular damage, widespread endothelial cell activation, platelet aggregates adhering to endothelial cells, and infiltrating macrophages and CD8+ T-cells.^99^

Endothelial damage is hypothesized to impact Long COVID,^181–184^ and multiple reports have described small fibrin-amyloid deposits in the bloodstream during acute COVID-19 that appear to persist in Long COVID.^135,185,186^ These so-named microclots can be induced in pre-pandemic control samples through exposure to S1 protein, suggesting that SARS-CoV-2 infection may trigger the accumulation of fibrin into an amyloid structure resistant to traditional protein degradation mechanisms. Kell and Pretorius hypothesize that these microclots impede only the smallest of capillaries, resulting in episodes of poor organ perfusion and reduced oxygen delivery and leaving delicate tissues prone to a well-established phenomenon called ischemic-reperfusion injury.^139,187^ Consistently, Akassoglou and colleagues recently showed that fibrin interacts directly with spike protein to form pro-inflammatory blood clots^106^ and promote neuron loss following infection through direct action on microglia after passing through the blood-brain barrier. In this study, monoclonal antibodies directed against the inflammatory fibrin domain protected mice against microglial activation and neuronal injury.^106^ These vascular changes may account for recent MRI findings in which post-COVID patients exhibited either significant hypoperfusion in the frontal lobe^188^ or widespread decreased brain oxygen levels.^189^ In primate models, SARS-CoV-2 infection induces microhemorrhages, brain hypoxia, and hypoxic-ischemic injury even in animals that did not develop severe respiratory disease, underscoring that neurological sequelae can readily occur following mild infections.^42^ As elevated fibrin levels are correlated with long-term cognitive deficits in patients who recovered from severe COVID-19,^190^ fibrin-targeted antibodies may be a potential therapeutic target for Long COVID patients with similar phenotypes.

Neurovascular coupling is a mechanism that regulates local cerebral blood flow in response to changes in neuronal activity. The nervous system has high energy demands which are broadly correlated with neuronal activity, and localized blood flow and blood glucose supply tend to increase during periods of high activity. Although this dynamic relationship is still not fully understood, it involves critical contributions from astrocytes and endothelial cells, cell types known to be highly impacted by SARS-CoV-2 infection (for detailed review, see ref.).^191^ Neurovascular coupling is difficult to measure in human patients, but glucose distribution and accumulation in the brain can be estimated in the clinic using fluorodeoxyglucose-positron emission tomography (FDG-PET). One longitudinal study in 56 adult Long COVID patients with persistent neurological symptoms found pronounced hypometabolism in the brainstem and cerebellum compared with healthy control participants, without significant changes between scans up to 32 months post-infection.^192^ These data suggest that the impact of COVID-19 infection on brain metabolism is relatively stable in Long COVID, without progressive worsening or improvement. Similarly, a retrospective FDG-PET study of 45 patients with prior COVID-19 infection found focal areas of hypometabolism in the cortex and cerebellum that peaked at 2 months and resolved within one year, suggesting that COVID-19 can induce temporary alterations in brain glucose metabolism even in patients who do not report Long COVID symptoms.^193^ In contrast, other FDG-PET studies of Long COVID report either higher cerebellar metabolism^194^ or no differences between Long COVID patients with primary fatigue and recovered controls.^195^ As FDG-PET only measures glucose distribution, these studies cannot provide further insight into a potential uncoupling of neuronal activity and blood flow in Long COVID.

## Central nervous system impacts of Long COVID

### Cortical changes and cognitive deficits

Cognitive deficits, often referred to by patients as “brain fog”, are commonly reported after SARS-CoV-2 infection.^2,3^ While patients who were hospitalized with severe disease have a high risk for prolonged cognitive dysfunction,^196–201^ several studies have also demonstrated lasting deficits in patients with mild or moderate infections.^202,203,203–211^ Self-reported cognitive deficits in the first 4 weeks following SARS-CoV-2 infection have been associated with the development of Long COVID symptoms,^212^ consistent with community cohort data showing that the largest deficits were observed in participants with unresolved persistent symptoms.^203^ Like most aspects of Long COVID, the long-term cognitive impacts of COVID-19 infection appear to be heterogeneous and may not be adequately measured by routine clinical exams; for example, a recent study reported no differences in cognitive function in Long COVID patients using the Single Digit Modalities Test, Stroop Task, and Trails A and B.^213^ However, more targeted assessments have revealed specific deficits in procedural memory and executive functioning in Long COVID patients. One NIH study examined the ability to acquire and consolidate new motor skills (typing tasks) over two training days to assess procedural memory formation. While early learning was comparable between groups, Long COVID patients showed significantly reduced overnight consolidation and slower overall typing speeds versus controls.^214^ Similarly, eye tracking studies from Long COVID patients with self-reported cognitive symptoms report significantly lower Montreal Cognitive Assessment scores and dysfunctional eye movement parameters (i.e. latency to movement, velocity, and accuracy of both visual and memory-guided saccades) that could be distinguished from healthy controls using machine learning.^215^

**Fig. 2 –:**
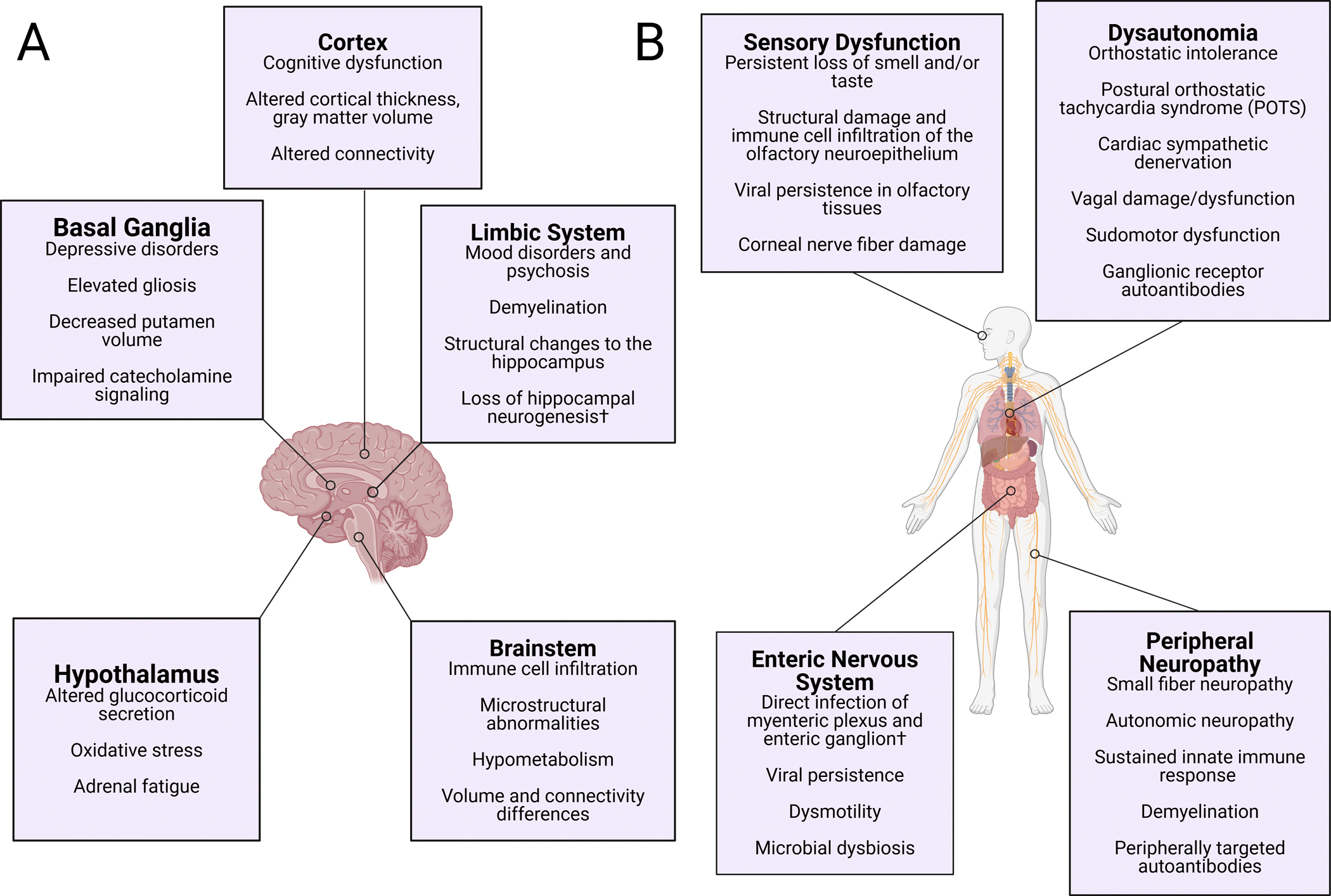
Neurological manifestations in A) the central nervous system and B) the peripheral nervous system in Long COVID. † denotes results from autopsy studies, animal models, or *in vitro* experiments that have not been directly measured in Long COVID. Figures generated using Biorender.

Deficits in cognitive and executive functioning implicate dysfunction of the cerebral cortex, the largest part of the human brain primarily responsible for coordinating complex thought processes (fig. 2A). MRI studies have identified alterations in cortical thickness, gray matter volume, and cerebral microstructure of COVID-19 patients across various cortical domains.^200,216–225^ Similar results have been reported in Long COVID, with sequelae such as anosmia and cognitive impairment showing associations with structural changes.^216,225–230^ One recent report used EEG to demonstrate decreased connectivity and increased disarray within cognitive structures following mild COVID-19.^231^ This study is particularly compelling as it took place in China during the first nationwide outbreak, meaning all participants were naïve to viral antigens and had roughly the same infection trajectories. Other MRI analyses found lower temporal and subcortical functional connectivity in non-hospitalized Long COVID patients,^232^ functional changes in the temporal lobe and superior parietal gyrus in COVID-19 survivors over 2 years post-infection,^233^ and hypoconnectivity in parahippocampal and orbitofrontal circuits in Long COVID patients after 1 year.^229^ The pathophysiology behind these changes is still unclear but could arise from disturbed balance between excitatory and inhibitory neurotransmission. Magnetic resonance spectroscopy of Long COVID patients has demonstrated higher levels of excitatory glutamate in the posterior cingulate cortex^234^ and lower levels of inhibitory GABA in the occipital cortex.^235^ In response to transcranial magnetic stimulation of the motor cortex, Long COVID patients showed a reduction of both long-interval intracortical inhibition (local suppression of motor cortex activity) and intracortical facilitation (excitatory activity within the motor cortex), while short-latency inhibition (suppression of motor potentials via sensory afferents) remained intact.^236^ Transcriptomic single-nuclei RNA sequencing has also revealed changes linked to synaptic deficits specifically within layer 2/3 excitatory neurons and inhibitory vasoactive intestinal peptide interneurons in the frontal cortex of COVID-19 decedents.^46^ As layer 2/3 excitatory neurons project to other cortical areas and are involved in associative learning,^237^ these data imply widespread dysregulation of cortical excitation in severe COVID-19. Overall, the data point toward increased disorganization of cortical circuitry in severe COVID-19 and potentially in Long COVID.

### Limbic system and basal ganglia

Neuropsychiatric symptoms are widely reported sequelae of SARS-CoV-2 infection, implicating involvement of limbic structures that regulate mood and emotion (fig. 2A). Several studies demonstrate higher incidence of psychiatric symptoms or new psychiatric diagnoses following COVID-19 which include major depression, anxiety disorders, and psychosis.^201,238–241^ Notably, measurements of anxiety and depression can be confounded by the presence of comorbid conditions like dysautonomia in Long COVID, which have overlapping symptoms that can artificially inflate scores in common self-reported symptom scales and lead to misdiagnosis.^242^ Although psychological and social factors undoubtedly play a role in the presentation of psychiatric symptoms, there is ample evidence that inflammation can directly influence behavioral and cognitive symptoms typically associated with mental health disorders. For example, major depressive disorder (MDD) is associated with chronic inflammation, and SSRI efficacy in depressed patients is correlated with the degree of reduction of peripheral inflammatory markers.^243^ Within the CNS, imaging for TSPO binding in patients with post-COVID depressive and cognitive symptoms has provided evidence for elevated gliosis and inflammation in the ventral striatum and dorsal putamen.^108^ These nuclei of the limbic system and basal ganglia have been linked to dysfunction in mental health disorders like major depression due to their roles in encoding motivational salience and initiating motor actions.^161,244–246^ In a 2024 follow-up study by the same group, the PET tracer [^11^C]SL25.1188 was used to estimate astrogliosis in a similar group of patients. PET signal was elevated in the prefrontal cortex, anterior cingulate, hippocampus, dorsal putamen, and ventral striatum of Long COVID patients versus controls.^110^ Another MRI study further implicates basal ganglia dysfunction in Long COVID with evidence that higher persistent symptom load was significantly associated with a smaller putamen volume alongside deficits in working memory, executive function, and recognition memory tasks.^247^ Furthermore, a recent MRI study reported extensive demyelination of projections between the limbic system and cortex that were the primary predictor of Hamilton depression scores and symptom severity in post-COVID patients.^113^ In rodent models, ICM injection of SARS-CoV-2 E protein was sufficient to induce depression-like behavior in the sucrose preference, tail suspension, and forced swim tests,^77^ and microglial cell density across the limbic system and basal ganglia exhibited overt accumulation 3 weeks after intranasal infection.^102^

The hippocampus is another highly specialized and intricately structured limbic region that plays a crucial role in memory consolidation, spatial navigation, and emotional processing.^248^ This region is particularly vulnerable to inflammation and neurodegeneration in conditions like seizure disorders and Alzheimer’s disease.^249^ Severe COVID-19 has been associated with significantly reduced left hippocampus thickness at 3 months post-infection,^250^ mirroring results from the longitudinal United Kingdom Biobank Study in which consistent abnormalities were found in the left parahippocampal gyrus of people reporting SARS-CoV-2 infection.^219^ In contrast, an early study from 2020 reported significantly higher bilateral gray matter volume in the hippocampus of COVID-19 patients 3 months after infection, which correlated with memory loss and anosmia.^251^ In humans the hippocampus is located proximally to the olfactory cortex, leading to speculation that neuroinvasion via the olfactory tract may predispose this region to damage by SARS-CoV-2. Histology studies of autopsy tissue have shown neuronal degeneration, glial activation, and structural changes in the hippocampus of COVID-19 decedents,^252–254^ and direct infusion of spike protein has been reported to induce neuronal cell death^79^ and synapse loss^78^ in mice. The hippocampus is also one of the rare sites of adult neurogenesis that may impact downstream cognitive processes,^255^ and decreased hippocampal neuroblast populations have been observed along with microglial activation in various COVID-19 rodent models.^105,111,256^ In Vanderheiden et al, the loss of hippocampal neurogenesis, elevations of IL-1β, and decreased novel object discrimination following infection of wild-type mice with the β.1.351 SARS-CoV-2 variant was abrogated by prior adenovirus vaccination against spike protein.

### Hypothalamus and HPA axis

The hypothalamus is a subcortical structure that participates in a wide array of bodily processes including temperature regulation, stress response, autonomic function, circadian rhythm, and satiety. Moreover, it is one of the anchors of the hypothalamic-pituitary-adrenal (HPA) axis, in which the hypothalamus exerts tight control over the pituitary gland to promote adrenal secretion of the glucocorticoid hormone cortisol to regulate stress responses and metabolism. Low morning serum cortisol has been identified as a significant predictor of Long COVID status in several patient cohorts,^64,257,258^ which may contribute to symptoms like fatigue and sleep disturbances. However, two other studies have since reported no differences in serum cortisol between Long COVID and control groups.^259,260^ Because cortisol follows a circadian pattern throughout the day,^261^ these discrepancies may arise from methodological differences in the time of sample collection. All of these studies to date have used a single timepoint, and further work is underway to determine how Long COVID impacts the diurnal pattern of corticosteroid secretion.

The hypothalamus is located adjacent to numerous circumventricular organs that make up the blood-CSF barrier and even harbors permeable fenestrated capillaries^262^ that could allow direct sampling of circulating plasma by hypothalamic cells.^263^ As SARS-CoV-2 appears to readily target and infect the choroid plexus, its proximity to the hypothalamus may predispose this region to localized neuroinflammation or even direct viral infection (fig. 2A). Infusion of S1 into the CSF, for example, is sufficient to induce inflammatory gene and cytokine expression in the hypothalamus of rats at both 24 hours and 7 days post-infusion.^81^ ICM S1 also potentiated subsequent inflammatory responses to LPS in rats, including a dramatic reduction in baseline brain corticosteroid levels.^82^ A similar study reported downregulation of nuclear factor erythroid 2-related factor 2 (Nrf2), a transcription factor described as a “master regulator” of inducible antioxidant and anti-inflammatory responses, in the paraventricular nucleus of the hypothalamus (PVN) one week after ICM S1 infusion.^80^ The PVN is heavily involved in the regulation of corticosteroid secretion through direct release of hormones into the bloodstream as well as projections to the pituitary, but also regulates processes such as fluid balance and sympathetic drive. Interestingly, Sun et al showed that rats treated with ICM S1 showed an exaggerated increase in mean daily blood pressure when treated with systemic angiotensin II which correlated with increased neuronal activation of the PVN, inflammatory gene expression, and Iba1+ microglial activation.^80^ Because the ICM S1 treatment prevented angiotensin II-induced activation of Nrf2, these data point toward spike-mediated disruption of anti-inflammatory signaling that impact homeostatic processes. Dysfunction of the PVN is known to cause hypersomnolence (excessive daytime sleepiness)^264^ and could contribute to the severe fatigue often reported in Long COVID.

### Brainstem

The brainstem is a critical area that regulates vital functions such as heart rate, respiration, and sensorimotor pathways. The brainstem also contains nuclei that project to essentially every other part of the brain, meaning that damage or dysfunction in these areas can have widespread impacts. SARS-CoV-2 infection does appear to directly impact the brainstem during acute infection (fig 2A): postmortem characterization of COVID-19 decedents reported robust microgliosis and axonal damage in the brainstem accompanied by invasion of CD8+ T cells, some B cells, and CD163+ perivascular macrophages.^96^ ACE2 and TMPRSS2 expression in the vagal nuclei of the medulla as well as the midbrain tegmentum^52^ suggest the potential for direct infection, and SARS-CoV-2 viral proteins have been found in the medulla and midbrain in a subset of COVID-19 decedents.^265^ However, a recent report found no evidence of N protein in CNS samples and that inflammatory type I interferon responses resolved in the later phase of the disease.^100^ Interestingly, transcriptomic analyses of those specimens found that brainstem endothelial cells, reactive microglia, and glutamatergic neurons still exhibited transcriptional changes consistent with ongoing inflammation, suggesting that direct infection is not necessary to drive immune-driven pathology. Indeed, a high-resolution MRI study of hospitalized patients found long-term microstructural abnormalities in the brainstem that persisted for months after infection.^266^ Functional studies of Long COVID patients have reported pronounced hypometabolism in the pons of 3 patients with post-COVID cognitive decline in addition to significantly larger brainstem and pons volume in Long COVID versus healthy controls.^267,268^ Another group used functional MRI during a Stoop color-word task to demonstrate brainstem connectivity differences in Long COVID.^269^ Interestingly, these connections were *stronger* in Long COVID, which Barnden et al speculate may reflect a compensatory response. The large overlap between Long COVID symptoms and brainstem processes highlights the need for further research to understand the long-term impacts of COVID-19 on this region.

## Peripheral nervous system and sensory dysfunction in Long COVID

### Autonomic nervous system (ANS) and dysautonomia

While originally described as part of the peripheral nervous system, the ANS is now more accurately referred to as the extended autonomic network that controls a wide swath of largely unconscious physiological processes geared toward maintaining body homeostasis: maintaining adequate blood volume and oxygen perfusion, regulating blood pressure and heart rate, controlling endocrine organs like sweat and salivary glands, regulating rates of digestion and excretion, among many others. Symptoms of dysautonomia vary widely and typically encompass multiple body systems, making these disorders particularly challenging to study with approaches historically focused on individual organ systems. Although no large study has systematically quantified differences in autonomic function in a large population of post-COVID patients, there is a general consensus from preliminary studies and case reports that dysautonomia is a relatively common outcome in COVID-19 (fig. 2B), with frequency estimates of specific cardiac autonomic dysfunctions ranging from 3–61%.^270,271^ Delayed diagnosis or misdiagnosis are commonly reported by dysautonomia patients^272,273^ and treatment options are quite limited, with both factors exacerbated by lack of accessible autonomic testing and blood-based biomarkers.

Orthostatic intolerance is frequently reported by Long COVID patients, which has brought renewed attention to a type of dysautonomia called postural orthostatic tachycardia syndrome (POTS). POTS is characterized by an exaggerated and sustained increase in heart rate when changing positions from supine to standing, accompanied by no change or an increase in blood pressure.^274^ As a result, patients with POTS frequently report exercise intolerance, fatigue, dizziness and altered consciousness, cognitive difficulties, and presyncope or syncope when upright; highly disabling symptoms that overlap substantially with Long COVID even in those who do not meet POTS criteria. One recent meta-analysis found POTS was twice as likely to be diagnosed in infected versus uninfected individuals with age as a significant covariable,^275^ suggesting that COVID-19 may act as a predisposing or initiating event for developing POTS. POTS has also been reported following routine COVID-19 vaccination in a large sample population, although the frequency of diagnosis was five times higher following infection versus vaccination in this study.^276^

Although estimated to impact up to 80% of Long COVID patients,^277^ the mechanisms underlying POTS and orthostatic intolerance in these patients remains largely unknown. There are likely several contributing factors such as decreased venous outflow and cerebral hypoperfusion, hypovolemia and fluid retention problems, heightened sympathetic activity, dysregulated peripheral catecholamine signaling, and autonomic neuropathy.^274^ Clinical autonomic testing is largely focused on cardiovascular and neuropathic measurements while the most common symptom scales (i.e. COMPASS-33) used as outcome measures were not developed or validated for use in POTS or Long COVID, making prevalence estimates difficult for specific POTS symptoms.^271^ A recent study used MIBG-SPECT for intravital imaging of myocardial sympathetic innervation and reported that 75% of patients with Long COVID referred for symptoms of dysautonomia had reduced MIBG uptake, suggestive of sympathetic denervation.^278^ Although this was a small study that lacked a control group, it indicates that ^123^I-MIBG SPECT may be a promising method to quantify sympathetic denervation and dysautonomia in an outpatient setting.

Post-COVID dysautonomia symptoms could arise from dysfunction within the vagus nerve, which is comprised of both sensory and motor neuron fibers that regulate control of the heart, lungs, digestive tract, and other systems. SARS-CoV-2 RNA and inflammatory cell infiltration of the vagus have been reported in COVID-19 decedents,^279^ and Long COVID patients are more likely to show thickening of the vagus measured by ultrasound compared with controls.^280^ Experiments in vagally-innervated mouse trachea-lung preparations have found that 49% of sensory fibers were activated by intratracheal S1 protein.^281^ The authors speculate that this direct activation of C-fibers may trigger symptoms like sneezing and coughing to further amplify viral spread, and similar dysfunction of vagal afferents could impact heart rate. Finally, norepinephrine release by vagal efferents to the spleen can stimulate T cells to release acetylcholine, which then acts on macrophages to inhibit inflammatory signaling.^282^ Notably, acetylcholine production by T cells is also required to control chronic viral infection in mice^283^ and regulate vasculature in humans.^284^ To our knowledge no study has directly examined this T-cell-dependent cholinergic anti-inflammatory pathway in Long COVID, although some case studies report a therapeutic effect of low-dose nicotine which may act by stimulating this pathway.^285^

One interesting hypothesis that was developed prior to the COVID-19 pandemic speculates that POTS patients develop autoantibodies against peripheral G-protein-coupled receptors, based on a handful of preliminary studies finding that POTS patients test positive for antibodies directed against adrenergic, muscarinic, and/or ganglionic cholinergic receptors in the ANS.^286–289^ As these receptors are principally responsible for neurotransmission in the peripheral ANS, autoantibodies could disrupt normal cardiovascular tone by acting as ligands, antagonists, or allosteric modulators.^290^ Particular attention has been drawn to autoantibodies against the α1 and β1 adrenergic receptors (ARs) in POTS, which could functionally disrupt baroreceptor-mediated vasoconstriction in response to standing.^289^ POTS phenotypes were observed in rabbits immunized against α1 and β1 ARs that were reversed following injection of antibody-neutralizing peptides, although this study did not use a dedicated control group.^291^ Interestingly, however, an exploratory study using traditional sandwich ELISA reported that Long COVID patients had an overall decrease in concentration of regulatory autoantibodies against GPCRs. When stratifying the data using a random forest classifier, the strongest classifiers were autoantibodies against α2 and β2 ARs along with the scavenger receptor stabilin-1; β2AR autoantibody levels also correlated with fatigue and vasomotor symptom severity.^292^ Although these studies are all limited by small sample sizes, these data highlight the possibility that post-COVID dysautonomia may be mechanistically distinct from non-COVID POTS while still converging on similar symptom profiles. Further development of animal models for post-COVID dysautonomia will be critical for dissecting potential receptor-based mechanisms of POTS symptoms.

### Chemosensory dysfunction

Olfactory dysfunction is a common symptom in acute COVID-19 and the most frequently encountered neurological manifestation of SARS-CoV-2 infection, with recent prevalence rates estimated to be as high as 68%.^293,294^ Although viral infections often cause transient smell loss, it seems particularly prevalent and prolonged post-COVID-19. For most individuals, these symptoms are transient and resolve within weeks.^293–295^ However, a significant minority experience lasting olfactory and chemosensory dysfunction (fig. 2B).^296,297^ In addition to impacting quality of life, chemosensory dysfunction can also have significant impacts on safety, such as being unable to detect smoke or spoiled food.^295^ As olfactory and chemosensory tracts involve direct connections from the CNS, these pathways have been the subject of intense research in COVID-19 pathology.

Indeed, multiple studies have reported that anosmia is associated with cognitive deficits,^298,299^ functional brain activity,^217^ cortical thinning,^216,217,227^ and structural damage,^216,227,300^ leading to speculation that the olfactory tract is an entry route for direct infection and/or immune cell recruitment. This possibility was supported by the detection of SARS-CoV-2 proteins in olfactory areas, including the primary olfactory cortex, of infected rhesus macaques.^57^ Histological examination of the olfactory neuroepithelium of 7 patients with COVID-19 and acute anosmia identified direct infection of sensory neurons, support cells, and immune cells that associated with local inflammation.^301^ Interestingly, viral transcripts and infected cells were also observed in samples from patients with long-term anosmia in this study, suggesting that viral persistence and inflammation of the olfactory epithelium may contribute to smell loss. However, a follow-up study by the same group recently reported that olfactory dysfunction was not dependent on neuroinvasion in hamsters,^302^ implicating alternative processes for sustained loss of smell. Consistently, an analysis of olfactory epithelial samples collected from patients with objectively quantified long-term smell loss from COVID-19 did not detect any viral RNA or protein, but instead reported diffuse infiltration of T cells, a shifted myeloid cell population, and inflammatory gene expression alongside a reduction in olfactory sensory neurons.^303^ Moreover, Peluso and colleagues recently used full-body PET imaging to examine the distribution of activated T cells in Long COVID. Compared with controls, Long COVID patients had significantly increased tracer uptake in many regions including the nasal turbinates, indicative of ongoing T cell infiltration of the olfactory system.^68^ Alternatively, a review paper by Butowt et al argues that, instead of directly infecting olfactory receptor neurons, SARS-CoV-2 predominately infects support cells (sustenacular cells) in the olfactory epithelium to cause rapid smell loss that largely recovers after regeneration of this population.^304^ In addition to sustenacular cells and macrophages in the olfactory epithelia, K18-hACE2 mice infected with SARS-CoV-2 showed direct infection of microglia and subpopulations of olfactory neurons within the piriform cortex and tubular striatum.^103^ The persistent olfactory impairment may be attributed to the widespread inflammation observed in the olfactory system and demyelination in olfactory pathways, as observed in animal models.^103^

### Mechanosensory deficits and peripheral neuropathy

Peripheral neuropathy, paresthesias, and other peripheral nerve symptoms are frequently reported following COVID-19 (fig. 2B).^305^ Many small studies have reported a high prevalence of small-fiber neuropathy (SFN) in Long COVID patients as measured by skin biopsy, often accompanied by sudomotor and autonomic dysfunction.^306–310^ Many viruses are associated with neuropathy and neuropathic pain, for instance, varicella zoster virus (VZV) can establish latency in dorsal root ganglia (DRG) neurons, with subsequent reactivation causing the disease shingles and accompanying herpetic neuralgia.^311^ Although SARS-CoV-2 is an RNA virus that is not traditionally thought to establish latent viral reservoirs, recent work is challenging this assumption.^70^ hiPSC-derived sensory neurons can be directly infected with SARS-CoV-2 *in vitro* but appear unable to produce infectious virus.^19^ However, rodent model studies have demonstrated viral proteins and particles in PNS neurons as well as replication within the trigeminal ganglion,^159,312^ indicating that direct infection and propagation within the PNS should not be ruled out.

Even in the absence of direct viral infection, systemic inflammation and viral byproducts can drive the development of SFN and peripheral neuropathies. In hamsters infected with intratracheal SARS-CoV-2, viral RNA was detected in DRGs within 24 hours of infection but no infectious material was found, suggesting that viral replication elsewhere in the body disseminates viral RNA to distal tissues.^313^ In this model, infected hamsters exhibited mechanical hypersensitivity that coincided with a “neuropathic transcriptome” in DRGs at 31 days post-infection, demonstrating a relationship between viral RNA dissemination and pain behavior. Interestingly, a follow-up study by the same group recently reported results from bulk RNA sequencing of the thoracic spinal cord in hamsters infected with SARS-CoV-2, influenza A, or control vehicle. While SARS-CoV-2 and influenza A appear to largely share transcriptional signatures, the SARS-CoV-2 group showed a unique enrichment of the extracellular matrix disassembly pathway at 31 days post-infection,^314^ which could explain prior data showing that influenza A virus does not cause the same mechanical hypersensitivity as SARS-CoV-2 in hamsters.^315^ Alternatively, muscle inflammation and pathology has been observed in Long COVID patients,^316^ which could potentially drive dysfunction in both motor neurons and sensory fibers. SARS-CoV-2 proteins may also interact directly with sensory receptors or their ligands: recent work has shown that cleaved spike protein has a high affinity for neuropilin 1 (NRP1), a receptor enriched in somatosensory pathways that may also function as a host entry receptor.^317^ In a rat model, spike protein decreased nociceptor activity and induced analgesia by inhibiting NRP1.^318^ The presence and duration of this analgesic effect in the context of COVID-19 infection is unknown, although a transient “silencing” of pain could potentially mask underlying neuropathic processes and account for the delayed onset of symptoms in Long COVID. Qualitative studies have also shown Long COVID is associated with a unique neuropathic phenotype, where patients describe feeling internal vibrations and tremors that are not accompanied by overt myoclonus (involuntary muscle twitches).^319,320^ Although the cause of these sensations is not known, it brings up the intriguing possibility that SARS-CoV-2 infection may directly impact sensory organs, such as the Pacinian corpuscles that sense vibration.

The role of autoimmunity in SFN is controversial, with no consistent pathogenic autoantibodies observed in general SFN patients.^321^ However, autoimmunity and immune dysfunction have also been proposed as major factors in post-COVID SFN, particularly in light of a recent retrospective cohort study by McAlpine et al demonstrating a positive clinical response to intravenous immunoglobulin therapy in 9 Long COVID patients with SFN as indicated by skin biopsies.^322^ While this study was limited to patient-reported outcomes, the high response rate nevertheless warrants further investigation as a therapeutic option for post-COVID SFN. In further support of a role of autoimmunity, recent preprints have reported that passive transfer of IgG from Long COVID patients to wildtype mice targets peripheral nerve tissue and drives pain phenotypes.^91,92^ Alternatively, sustained myeloid cell activation and proinflammatory cytokines may contribute to SFN phenotypes.^323^ Soluble inflammatory mediators are known to drive nociceptor activity,^324^ and activated myeloid cells could contribute to peripheral nerve damage. Indeed, patients with neurological symptoms 4 weeks after COVID-19 exhibited lower corneal nerve fiber density and fiber length by intravital corneal confocal microscopy.^325^ Long COVID patients without neurological symptoms did not have overt fiber abnormalities, but importantly, both groups of patients had elevated numbers of corneal dendritic cells compared with controls, suggesting ongoing leukocyte infiltration of the PNS post-COVID-19.

### Enteric nervous system

Gastrointestinal (GI) complaints are common in both acute COVID-19 and Long COVID, with one meta-analysis estimating prevalence to be 12% after acute infection and 22% in Long COVID cohorts.^326^ Symptoms like dysmotility and gastroparesis implicate involvement of the nervous system in at least some GI presentations (fig. 2B). The majority of research focused on the GI system in COVID-19 has focused on intestinal pathology rather than the enteric nervous system.^327^ ACE2 and TMPRSS2 are expressed in enteric neurons and glial cells of the small and large intestine, suggesting these cell types may be susceptible to viral entry.^172^ As SARS-CoV-2 replicates within the GI tract,^328^ it is plausible that enteric neurons and glia are highly exposed and therefore vulnerable to direct infection. Indeed, strong labeling of neurons in the myenteric plexus and enteric ganglion for SARS-CoV-2 N protein in COVID-19 decedents is strongly indicative of direct SARS-CoV-2 infection,^329^ and full-body PET imaging showed widespread accumulation of activated T cells in the GI tract of Long COVID patients.^68^ Some researchers speculate that the GI tract may be a site of long-term antigen persistence, in which case lingering spike protein or viral RNA could drive further dysfunction of the enteric nervous system.^68^ Changes to the gut microbiome have also been implicated in Long COVID.^330^ Fecal transplants from Long COVID patients to germ-free mice blocked novel object discrimination and increased TNF transcript levels in the hippocampus, demonstrating that alterations in the gut microbiome can drive CNS inflammation and behavioral phenotypes.^331^

## Interactions with neurodegenerative diseases

Although the long-term effects of SARS-CoV-2 infection are still unknown, there is potential for exacerbation of neurodegenerative diseases such as Parkinson’s disease (PD), as has been suggested for other infections (fig. 1).^332^ PD is characterized by motor and gait disturbances arising from degeneration of dopaminergic neurons in the substantia nigra (SN),^333^ which are particularly sensitive to inflammation and oxidative stress.^161,246,334^ An early report published in 2020 found that both motor and non-motor symptoms were significantly worsened in PD patients who experienced a COVID-19 infection,^335^ and a recent cross-sectional study reported that SARS-CoV-2 infection contributes to the deterioration of motor symptoms and promotes increased susceptibility to COVID-19.^336^ SARS-CoV-2 has been shown to infect human iPSC-derived dopamine neurons *in vitro*, triggering α-synuclein accumulation, cellular senescence, and cell death.^55,157^ A similar profile of gene expression suggesting cellular senescence was also found in the SN of patients who died from COVID-19.^55^ In mice expressing human ACE2, injection of preformed α-synuclein fibrils exacerbated neuroinflammation in the substantia nigra following SARS-CoV-2 infection and led to loss of dopamine neurons.^157^ Additionally, a recent preprint found that mice infected with murine adapted SARS-CoV-2 showed degeneration of dopamine neurons in the olfactory bulb and SN, supporting a mechanistic link between COVID-19 and PD progression.^156^ Taken together, these data suggest that neuroinflammation induced by COVID-19 could enhance progression of PD and worsen outcomes for patients.

SARS-CoV-2 may also impact the initiation or progression of Alzheimer’s Disease (AD), a progressive dementia associated with the accumulation of amyloid precursor protein (APP) and hyperphosphorylated tau protein (fig. 1). One early autopsy study reported increased APP deposits in the brainstem of COVID-19 patients versus controls,^96^ while another identified microglia and astrocyte subpopulations that shared features with those reported in AD.^46^ Another comparison of tissue from patients with AD, SARS-CoV-2, AD with SARS-CoV-2, and healthy controls found similar profiles of neuroinflammation and microvascular injury across AD and SARS-CoV-2 patients alongside nodular formation in the SARS-CoV-2 infected AD group, suggesting infection can exacerbate neuroinflammation and AD progression.^337^ Similarly, an independent postmortem analysis found exacerbated microgliosis and dysregulation of oligodendrocyte gene expression in the hippocampus of AD patients that persisted for months post-infection.^338^ Consistently, a recent report using the UK Biobank found elevated AD-associated biomarkers following COVID-19, which were linked to structural imaging patterns and cognitive dysfunction.^339^

While it seems likely that SARS-CoV-2 infection impacts the progression of AD, it is unclear if infection can directly precipitate AD in previously healthy patients. The most direct evidence in support of AD initiation comes from a recent study of postmortem brain samples from individuals who died of unrelated causes 4–13 months after acute COVID-19. Phosphorylated tau was upregulated in this clinically recovered group compared with uninfected controls and individuals who died with COVID-19, while activation of microglia and inflammatory cytokines expression were consistent between acute and recovered COVID-19.^340^ Hospitalization with COVID-19 was associated with a higher risk of AD alongside a significant positive genetic correlation in one Mendelian randomization study,^341^ although a different 2-sample Mendelian randomization analysis reported that genetic susceptibility to COVID-19 was not associated with risk or progression of AD, suggesting that a relationship between COVID-19 and AD may be independent of illness severity.^342^ As most studies have focused on hospitalized or critically ill patients, the impact of mild or repeated infections on AD risk remains unknown. A study examining CSF from a large cohort of Long COVID patients reported no observable AD-related pathology and suggests that cognitive impairment in Long COVID may be distinct from AD, at least in the months or years immediately following infection.^202^

## Summary and concluding remarks

Despite the perception that COVID-19 is now a mild disease, there is overwhelming evidence indicating that SARS-CoV-2 infection is capable of producing widespread post-acute sequelae in a significant percentage of infections. This includes a substantial impact on the nervous system resulting from a combination of direct infection, systemic inflammation, immune dysfunction, vascular complications, and tissue hypoxia. These factors range from changes in neurotransmitter metabolism to blood-brain barrier dysfunction. Both innate and adaptive immune responses appear to play a role in the pathophysiology of Long COVID by giving rise to pronounced neuroinflammation, synaptic remodeling, infiltration of CNS parenchyma, and autoimmunity. Understanding the pathophysiology of Long COVID will require a focus on neuroimmune interactions and continued investment in mechanistic research. Unfortunately, there are no treatments or therapies that have proven effective for Long COVID, and the highly heterogeneous nature of the condition means a personalized treatment approach will likely be necessary. Overall, these data highlight the need for better approaches to both treat and prevent Long COVID.
